# Supplementary material for: DeePathology: Deep Multi-Task Learning for Inferring Molecular Pathology from Cancer Transcriptome
Source: Sci Rep. 2019 Nov 11;9:16526. doi: 10.1038/s41598-019-52937-5 (PMC6848155; doi:10.1038/s41598-019-52937-5)
Supplement: Supplementary file 1 — Supplementary Information [file 41598_2019_52937_MOESM1_ESM.pdf]

Supplementary Information: DeePathology: Deep Multi-Task Learning for Inferring Molecular Pathology from Cancer Transcriptome

Behrooz Azarkhalili[1,4], Ali Saberi[2], Hamidreza Chitsaz[3], and Ali Sharifi-Zarchi[2, *]

[1]Department of Stem Cell Biology and Technology, Royan Institute, Tehran, Iran
[2]Department of Computer Engineering, Sharif University of Technology, Tehran, Iran
[3]Department of Computer Science, Colorado State University, Fort Collins, CO, USA
[4]Department of Mathematics and Computer Science, Sharif University of Technolog, Tehran, Iran
[*]Corresponding author: asharifi@sharif.edu

Table S1: The number of samples used for each tissue type.

	GDC Data			DeePathology 10750 Selection			5-Fold Cross Validation Selection			5-Fold Cross Validation Selection (Folds #1 to #4)			5-Fold Cross Validation Selection (Fold #5)		
**Tissue**	**Tumor**	**Normal**	**Total**	**Tumor**	**Normal**	**Total**	**Train**	**Test**	**Total**	**Train**	**Test**	**Total**	**Train**	**Test**	**Total**
Adrenal Gland	262	3	265	262	3	265	212	53	265	212	53	265	212	53	265
Bile Duct	36	9	45	36	9	45	36	9	45	36	9	45	36	9	45
Bladder	411	19	430	411	19	430	344	86	430	344	86	430	344	86	430
Blood	126	0	126	126	0	126	101	25	126	101	25	126	100	26	126
Bone Marrow	83	0	83	83	0	83	67	16	83	67	16	83	64	19	83
Brain	525	0	525	525	0	525	420	105	525	420	105	525	420	105	525
Breast	1088	104	1192	1088	104	1192	954	238	1192	954	238	1192	952	240	1192
Cervix	306	3	309	306	3	309	248	61	309	248	61	309	244	65	309
Colorectal	611	12	623	611	12	623	499	124	623	499	124	623	496	127	623
Esophagus	162	11	173	162	11	173	139	34	173	139	34	173	136	37	173
Eye	80	0	80	80	0	80	64	16	80	64	16	80	64	16	80
Head and Neck	493	44	537	493	44	537	430	107	537	430	107	537	428	109	537
Kidney	964	141	1105	964	141	1105	884	221	1105	884	221	1105	884	221	1105
Liver	370	50	420	370	50	420	336	84	420	336	84	420	336	84	420
Lung	987	59	1046	987	59	1046	837	209	1046	837	209	1046	836	210	1046
Lymph Nodes	47	0	47	47	0	47	38	9	47	38	9	47	36	11	47
Ovary	376	0	376	376	0	376	301	75	376	301	75	376	300	76	376
Pancreas	178	4	182	178	4	182	146	36	182	146	36	182	144	38	182
Pleura	86	0	86	86	0	86	69	17	86	69	17	86	68	18	86
Prostate	495	52	547	495	52	547	438	109	547	438	109	547	436	111	547
Skin	449	1	450	449	1	450	360	90	450	360	90	450	360	90	450
Soft Tissue	261	0	261	261	0	261	209	52	261	209	52	261	208	53	261
Stomach	372	32	404	372	32	404	324	80	404	324	80	404	320	84	404
Testis	156	0	156	156	0	156	125	31	156	125	31	156	124	32	156
Thymus	119	2	121	119	2	121	97	24	121	97	24	121	96	25	121
Thyroid	509	58	567	509	58	567	454	113	567	454	113	567	452	115	567
Uterus	598	33	631	572	22	594	476	118	594	476	118	594	472	122	594
**All**	**10150**	**637**	**10787**	**10124**	**626**	**10750**	**8608**	**2142**	**10750**	**8608**	**2142**	**10750**	**8568**	**2182**	**10750**

Table S2: The number of samples used for each cancer type.

				GDC Data	DeePathology 10750 Selection	5-Fold Cross Validation Selection			5-Fold Cross Validation Selection (Folds #1 to #4)			5-Fold Cross Validation Selection (Fold #5)		
**Disease**	**Code**	**Project**	**Tissue**	**Total**	**Total**	**Train**	**Test**	**Total**	**Train**	**Test**	**Total**	**Train**	**Test**	**Total**
Acute Myeloid Leukemia	LAML	TCGA-LAML + TARGET-AML	Bone Marrow + Blood	209	209	168	41	209	168	41	209	164	45	209
Adrenocortical Carcinoma	ACC	TCGA-ACC	Adrenal Gland	79	79	64	15	79	64	15	79	60	19	79
Bladder Urothelial Carcinoma	BLCA	TCGA-BLCA	Bladder	411	411	329	82	411	329	82	411	328	83	411
Brain Lower Grade Glioma	LGG	TCGA-LGG	Brain	525	525	420	105	525	420	105	525	420	105	525
Breast Invasive Carcinoma	BRCA	TCGA-BRCA	Breast	1088	1088	871	217	1088	871	217	1088	868	220	1088
Cervical Squamous Cell Carcinoma and Endo	CESC	TCGA-CESC	Cervix	306	306	245	61	306	245	61	306	244	62	306
Cholangiocarcinoma	CHOL	TCGA-CHOL	Bile Duct	36	36	29	7	36	29	7	36	28	8	36
Colon Adenocarcinoma	COAD	TCGA-COAD	Colorectal	451	451	361	90	451	361	90	451	360	91	451
Esophageal Carcinoma	ESCA	TCGA-ESCA	Esophagus	162	162	130	32	162	130	32	162	128	34	162
Head and Neck Squamous Cell Carcinoma	HNSC	TCGA-HNSC	Head and Neck	493	493	395	98	493	395	98	493	392	101	493
High-Risk Wilms Tumor	WITU	TARGET-WT	Kidney	122	122	98	24	122	98	24	122	96	26	122
Kidney Chromophobe	KICH	TCGA-KICH	Kidney	65	65	52	13	65	52	13	65	52	13	65
Kidney Renal Clear Cell Carcinoma	KIRC	TCGA-KIRC	Kidney	492	492	394	98	492	394	98	492	392	100	492
Kidney Renal Papillary Cell Carcinoma	KIRP	TCGA-KIRP	Kidney	285	285	228	57	285	228	57	285	228	57	285
Liver Hepatocellular Carcinoma	LIHC	TCGA-LIHC	Liver	370	370	296	74	370	296	74	370	296	74	370
Lung Adenocarcinoma	LUAD	TCGA-LUAD	Lung	512	512	410	102	512	410	102	512	408	104	512
Lung Squamous Cell Carcinoma	LUSC	TCGA-LUSC	Lung	475	475	380	95	475	380	95	475	380	95	475
Lymphoid Neoplasm Diffuse Large B-cell Lyn	DLBC	TCGA-DLBC	Lymph Nodes	47	47	38	9	47	38	9	47	36	11	47
Mesothelioma	MESO	TCGA-MESO	Pleura	86	86	69	17	86	69	17	86	68	18	86
Normal	Normal	Normal Samples of All Projects	All	637	626	501	125	626	501	125	626	500	126	626
Ovarian Serous Cystadenocarcinoma	OV	TCGA-OV	Ovary	376	376	301	75	376	301	75	376	300	76	376
Pancreatic Adenocarcinoma	PAAD	TCGA-PAAD	Pancreas	178	178	143	35	178	143	35	178	140	38	178
Pheochromocytoma and Paraganglioma	PCPG	TCGA-PCPG	Adrenal Gland	183	183	147	36	183	147	36	183	144	39	183
Prostate Adenocarcinoma	PRAD	TCGA-PRAD	Prostate	495	495	396	99	495	396	99	495	396	99	495
Rectum Adenocarcinoma	REAd	TCGA-READ	Colorectal	160	160	128	32	160	128	32	160	128	32	160
Sarcoma	SARC	TCGA-SARC	Soft Tissue	261	261	209	52	261	209	52	261	208	53	261
Skin Cutaneous Melanoma	SKCM	TCGA-SKCM	Skin	449	449	360	89	449	360	89	449	356	93	449
Stomach Adenocarcinoma	STAD	TCGA-STAD	Stomach	372	372	298	74	372	298	74	372	296	76	372
Testicular Germ Cell Tumors	TGCT	TCGA-TGCT	Testis	156	156	125	31	156	125	31	156	124	32	156
Thymoma	THYM	TCGA-THYM	Thymus	119	119	96	23	119	96	23	119	92	27	119
Thyroid Carcinoma	THCA	TCGA-THCA	Thyroid	509	509	408	101	509	408	101	509	404	105	509
Uterine Carcinosarcoma	UCS	TCGA-UCS	Uterus	56	56	45	11	56	45	11	56	44	12	56
Uterine Corpus Endometrial Carcinoma	UCEC	TCGA-UCEC	Uterus	542	516	413	103	516	413	103	516	412	104	516
Uveal Melanoma	UVM	TCGA-UVM	Eye	80	80	64	16	80	64	16	80	64	16	80
**All**				**10787**	**10750**	**8611**	**2139**	**10750**	**8611**	**2139**	**10750**	**8556**	**2194**	**10750**

Table S3: Sensitivity, specificity, F1 metric and balanced accuracy of DNN classification for each tissue.

Tissue	Sensitivity	Specificity	F1	Balanced Accuracy
Adrenal Gland	0.975	1	0.964	0.987
Bile Duct	0.878	0.756	0.857	0.817
Bladder	0.965	0.959	0.993	0.962
Blood	1	1	1	1
Bone Marrow	0.988	1	0.994	0.994
Brain	1	1	1	1
Breast	0.998	0.992	0.991	0.995
Cervix	0.977	0.951	0.982	0.964
Colorectal	0.998	0.996	0.991	0.997
Esophagus	0.913	0.965	0.916	0.924
Eye	0.991	0.997	0.988	0.994
Head and Neck	0.989	0.965	0.981	0.977
Kidney	0.997	0.995	0.997	0.996
Liver	0.992	0.976	0.981	0.984
Lung	0.984	0.99	0.986	0.987
Lymph Nodes	1	1	1	1
Ovary	0.999	1	0.992	0.999
Pancreas	0.984	0.976	0.978	0.98
Pleura	0.977	0.965	0.988	0.971
Prostate	1	1	1	1
Skin	0.98	0.992	0.987	0.986
Soft Tissue	0.977	0.963	0.971	0.97
Stomach	0.96	0.972	0.962	0.966
Testis	1	1	1	1
Thymus	0.997	0.987	0.989	0.992
Thyroid	1	0.998	0.998	0.999
Uterus	0.991	0.995	0.992	0.993

Table S4: Sensitivity, specificity, F1 metric and balanced accuracy of DNN classification for each disease type.

Disease	Sensitivity	Specificity	F1	Balanced Accuracy
LAML	0.939	0.998	0.964	0.968
ACC	1	1	1	1
BLCA	0.986	1	0.981	0.993
LGG	0.718	0.999	0.811	0.859
BRCA	0.9	0.991	0.882	0.946
CESC	0.854	0.998	0.847	0.926
CHOL	0.994	1	0.992	0.997
COAD	0.95	0.997	0.954	0.973
ESCA	0.883	0.999	0.864	0.941
HNSC	0.943	0.998	0.934	0.971
WITU	0.966	0.999	0.974	0.981
KICH	0.922	0.995	0.91	0.958
KIRC	0.893	0.991	0.883	0.944
KIRP	1	1	1	1
LIHC	0.943	1	0.923	0.971
LUAD	0.836	0.991	0.858	0.913
LUSC	0.995	1	0.989	0.997
DLBC	0.966	0.995	0.949	0.982
MESO	0.513	0.996	0.556	0.754
Normal	0.929	0.998	0.936	0.958
OV	0.978	1	0.956	0.989
PAAD	0.975	0.999	0.951	0.987
PCPG	0.936	1	0.912	0.968
PRAD	0.943	0.999	0.94	0.97
READ	0.975	1	0.965	0.987
SARC	0.93	0.997	0.91	0.964
SKCM	0.976	0.999	0.964	0.987
STAD	1	1	1	1
TGCT	0.991	1	0.983	0.997
THYM	0.982	0.999	0.986	0.991
THCA	0.797	0.999	0.743	0.898
UCS	0.976	0.998	0.964	0.987
UCEC	0.991	1	0.989	0.994
UVM	0.955	0.998	0.935	0.977

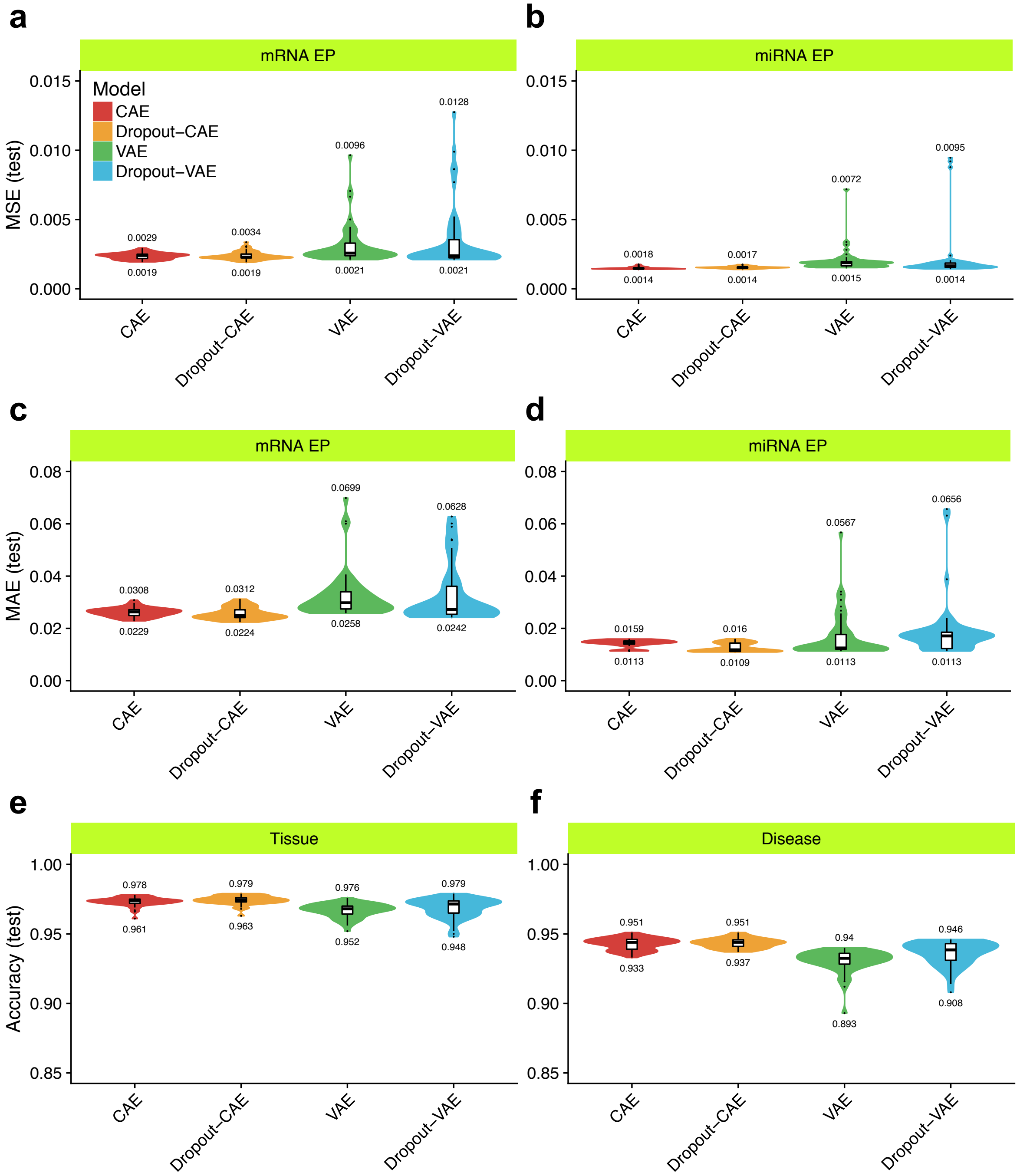

Figure S1: Distribution of different error or accuracy measurements during hyperparameter optimization: **(a)** mean square error (MSE) of reproducing mRNA expression profiles (EP), **(b)** MSE of generating miRNA expression profiles, **(c)** mean absolute error (MAE) of reproducing mRNA EP, **(d)** MAE of reproducing miRNA EP, **(e)** accuracy of predicting tissue for the test dataset, **(f)** accuracy of predicting cancer type for the test dataset. In each violin plot, the colors represent different architectures.

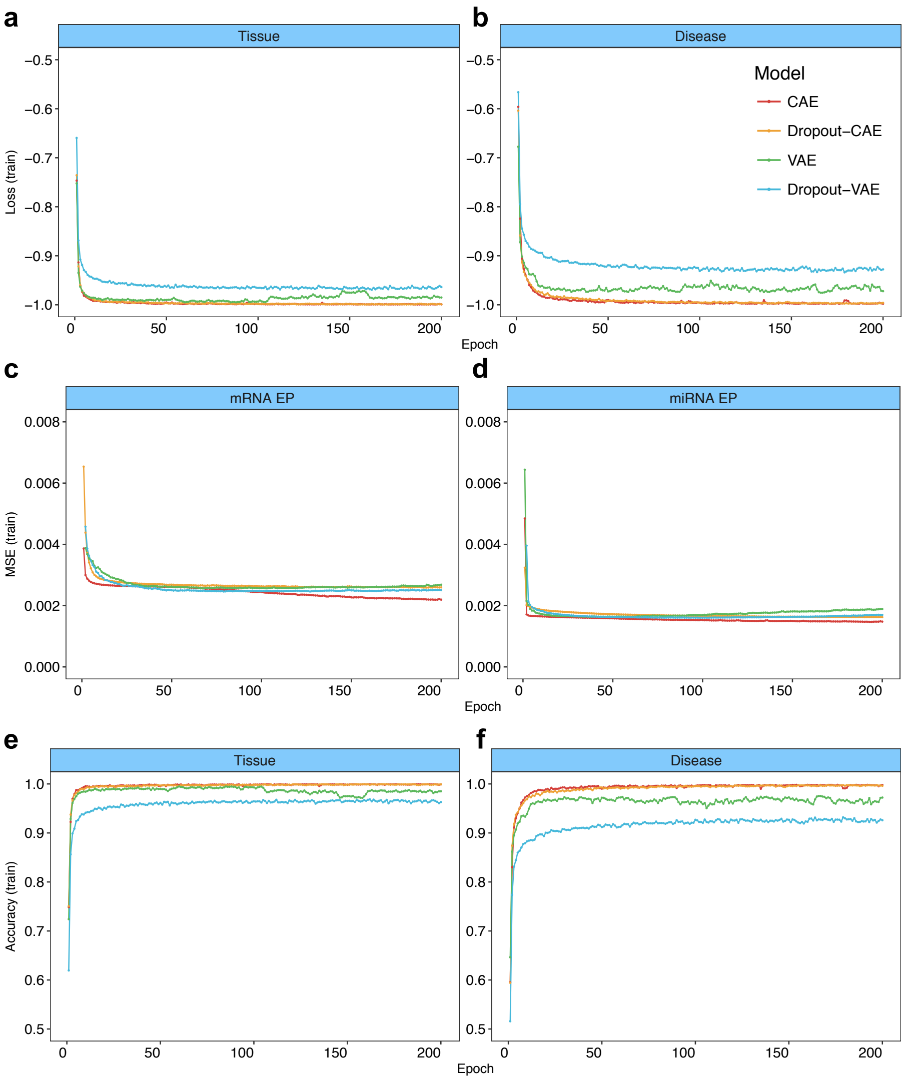

Figure S2: Performance of DNNs on training data, during 200 epochs of training. In each plot, the x-axis shows the training epochs, and the y-axis shows: **(a)** the value of loss function for predicting tissue type, **(b)** the value of loss function for predicting disease state, **(c)** mean square error (MSE) of reproducing mRNA expression profiles (EP), **(d)** MSE of predicting miRNA EP, **(e)** accuracy of predicting tissue, and **(f)** accuracy of predicting cancer type. All results are based on the training dataset.

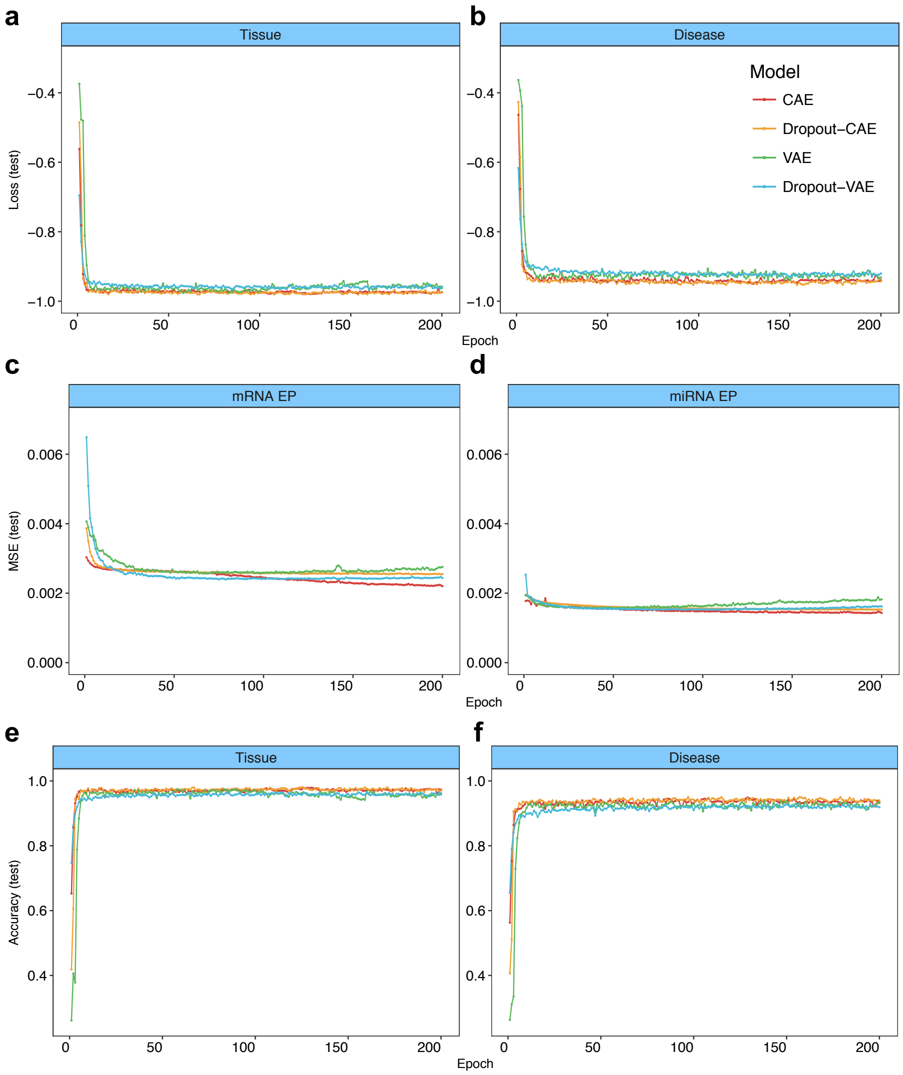

Figure S3: Performance of DNNs on test data, during 200 epochs of training. In each plot, the x-axis shows the training epochs, and the y-axis shows: **(a)** the value of loss function for predicting tissue type, **(b)** the value of loss function for predicting disease state, **(c)** mean square error (MSE) of reproducing mRNA expression profiles (EP), **(d)** MSE of predicting miRNA EP, **(e)** accuracy of predicting tissue, and **(f)** accuracy of predicting cancer type. All results are based on the test dataset.

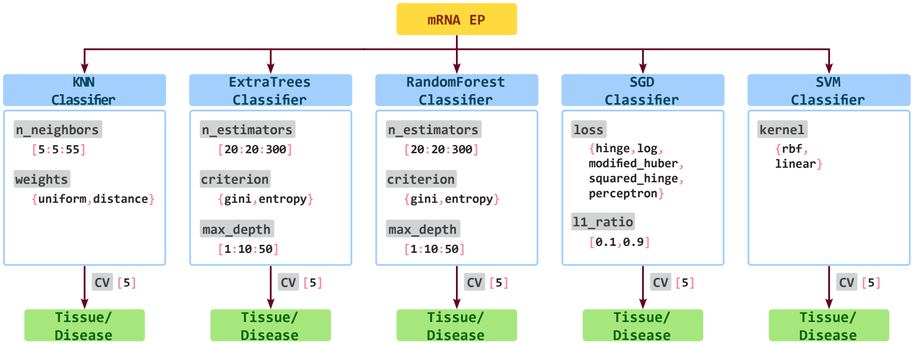

Figure S4: Hyperparameter optmization of the other classification algorithms. Here the notation $[start : step : end]$ return evenly spaced values within the close interval $[start, stop]$ with increments equal to $step$. A dictionary $\{a, b, c\}$ means that all of the item a, b, and c can be selected in the Bayesian optimization process.

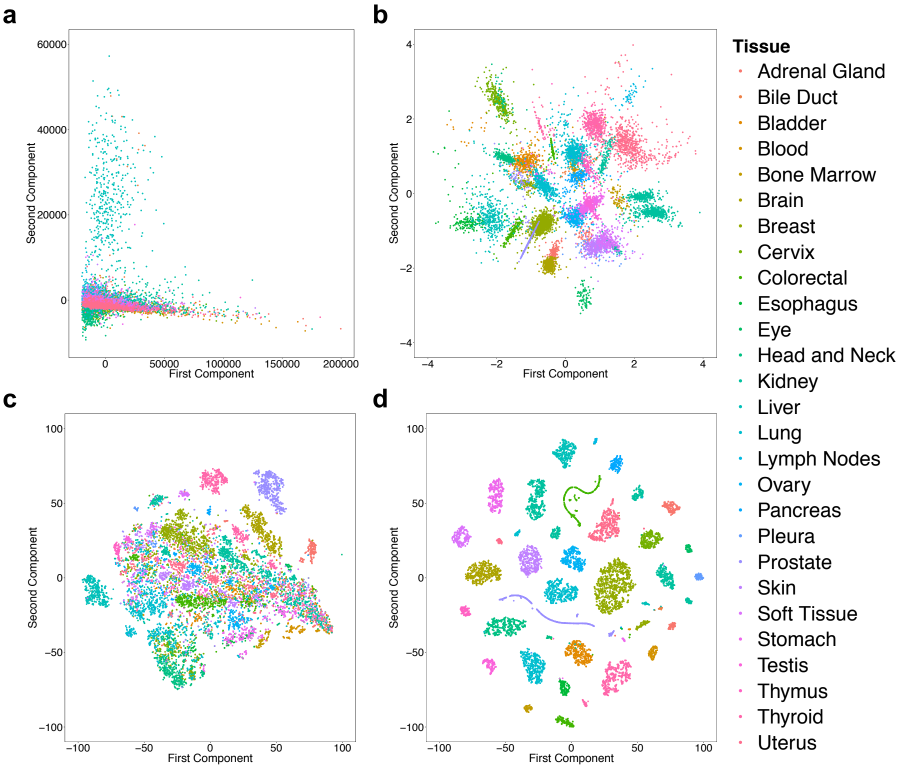

Figure S5: Discrimination of sample tissues in the original and Cell Identity Codes (CIC) spaces **(a)** PCA plot of the original mRNA expression profiles of all samples. Each dot and its color show a sample and its tissue type, respectively. **(b)** PCA plot of the 8-dimensional CIC space. **(c)** t-SNE plot of the original mRNA expression profiles. **(d)** t-SNE plot of the 8-dimensional CIC space.
